# Pathogenic landscapes: Interactions between land, people, disease vectors, and their animal hosts

**DOI:** 10.1186/1476-072X-9-54

**Published:** 2010-10-27

**Authors:** Eric F Lambin, Annelise Tran, Sophie O Vanwambeke, Catherine Linard, Valérie Soti

**Affiliations:** 1 Georges Lemaître Centre for Earth and Climate Research, Earth and Life Institute, University of Louvain, 3 place Pasteur, Louvain-la-Neuve, B-1348, Belgium; 2 School of Earth Sciences and Woods Institute, Stanford University, 473 Via Ortega, Stanford, CA 94305-4216, USA; 3 CIRAD, Animal et gestion intégrée des risques (Agirs), CIRAD, Montpellier, France; 4 CIRAD, UMR Territoires, environnement, télédétection et information spatiale (TETIS), CIRAD, Montpellier, France; 5 SAS Nevantropic, Cayenne, French Guiana, France; 6 Spatial Ecology and Epidemiology Group, Department of Zoology, University of Oxford, Tinbergen Building, South Parks Road, Oxford, OX1 3PS, UK

## Abstract

**Background:**

Landscape attributes influence spatial variations in disease risk or incidence. We present a review of the key findings from eight case studies that we conducted in Europe and West Africa on the impact of land changes on emerging or re-emerging vector-borne diseases and/or zoonoses. The case studies concern West Nile virus transmission in Senegal, tick-borne encephalitis incidence in Latvia, sandfly abundance in the French Pyrenees, Rift Valley Fever in the Ferlo (Senegal), West Nile Fever and the risk of malaria re-emergence in the Camargue, and rodent-borne Puumala hantavirus and Lyme borreliosis in Belgium.

**Results:**

We identified general principles governing landscape epidemiology in these diverse disease systems and geographic regions. We formulated ten propositions that are related to landscape attributes, spatial patterns and habitat connectivity, pathways of pathogen transmission between vectors and hosts, scale issues, land use and ownership, and human behaviour associated with transmission cycles.

**Conclusions:**

A static view of the "pathogenecity" of landscapes overlays maps of the spatial distribution of vectors and their habitats, animal hosts carrying specific pathogens and their habitat, and susceptible human hosts and their land use. A more dynamic view emphasizing the spatial and temporal interactions between these agents at multiple scales is more appropriate. We also highlight the complementarity of the modelling approaches used in our case studies. Integrated analyses at the landscape scale allows a better understanding of interactions between changes in ecosystems and climate, land use and human behaviour, and the ecology of vectors and animal hosts of infectious agents.

## Introduction

The emergence and re-emergence of vector-borne and zoonotic diseases is controlled by ecosystem changes at the landscape level, in addition to other factors [1]. Spatial (or landscape) epidemiology is defined as the study of spatial variation in disease risk or incidence [2]. An integrated analysis at the landscape scale allows a better understanding of interactions between changes in ecosystems and climate, land use and human behaviour, and the ecology of vectors and animal hosts of infectious agents. Although Hippocrates already recognized the importance of the environment on health, scientists such as Jacques May and Eugene Pavlovsky formalized these ideas in the 20^th ^century. Medical geography was defined as the study of the distribution of manifested and potential diseases over the earth's surface followed by the study of correlations between these and environmental factors [3]. The Russian epidemiologist Pavlovsky coined the term "landscape epidemiology":

*Figuratively speaking, the existence of the natural focus of a transmissible disease depends on a continuous interaction of the quintet (five) of its prerequisites associated with a specific geographic landscape *[4].

These five prerequesites were listed as:

*(1) Animal donors; (2) vectors; (3) animal recipients; (4) the pathogenic agent itself in an infective state, (5) the influence of factors of the external environments contributing to an unhindered transmission of infection from one organism to another (circulation of pathogenic agent) *[4].

Spatial interactions between these agents in a landscape explain patterns of infection risk and may contribute to disease emergence. Analysing these complex landscape systems of interacting agents requires an interdisciplinary approach. Data from different sources and collected at different scales need to be linked, using innovative analytic methods.

As part of a broader project on the impact of environmental changes on vector-borne diseases, we conducted a series of landscape scale studies of different disease systems in Europe and West Africa. These studies allowed better understanding of the influence of landscapes on the transmission of each of these infections. Here we present a review of the key findings from these case studies on the impact of land changes on emerging or re-emerging diseases that are transmitted by arthropod vectors and/or have an animal origin (zoonoses). Through an inductive approach, we identify general principles governing spatial epidemiology, outlining a set of propositions of general validity for different diseases and geographic contexts. All these propositions are related to spatial patterns and processes associated with transmission cycles at the landscape scale. Together, they contribute to advance the theory of spatial epidemiology.

## Methods

### Analytic tools used for the case studies

We developed various methods to understand interactions between land change, vectors, animal and human hosts. It was crucial to develop tools to link the various components of disease systems across space and to model spatial interactions. These innovations in analytic methods were a key component for developing an integrated approach of disease systems. For each site, we produced detailed land cover maps based on remote sensing data at medium to fine spatial resolutions and extracted landscape metrics. We also mapped land surface brightness, greenness, and wetness based on these data. Various spatial statistical models were used. Habitat suitability models relate the presence or abundance of vectors or animal hosts to landscape predictors that represent aspects of the species' habitat- e.g., vegetation cover, landscape configuration, surface moisture, topography, soil types. The spatial units for these models were small plots for which field observations on vector presence or abundance were collected. A variant models infection prevalence among vectors and/or hosts, as measured by field trapping in different landscapes. Another type of spatial statistical models used human cases, as compiled by public health records. Explanatory variables in these models are demographic and socio-economic variables from census data, land use maps including types of settlements, proxy variables for risk behaviours, climate and land cover. The spatial units of these latter models are administrative units at which census data are aggregated.

The Basic Reproductive Rate (R_0_) - which quantifies the average number of new infections that will arise from introducing an infective host into a susceptible population [5] - has been extensively used [6,7]. R_0 _is generally estimated at the aggregate level of populations. In estimating R_0_, the degree of contact between people and vectors is an essential factor for disease dynamics. Factors influencing people-vector contacts include the relative population densities and spatial distributions of both vectors and people [2], and their movements and behaviours. Previous studies based on R_0 _have often assumed a constant value across space of the human biting rate, given the difficulty in obtaining spatially-explicit and quantitative estimates of this variable. By coupling R_0 _models with spatial statistical models, we spatialized R_0 _and therefore better represented the spatial heterogeneity in the risk of establishment of an infection. Among the various input variables forming R_0_, the vector-host ratio displays the greatest spatial heterogeneity. By combining fine-scale land cover variables and coarse-scale climatic variables, we predicted the spatial distribution of vectors. This was then integrated with maps of human host distribution to spatialize the vector-host ratio in the R_0 _formula [8,9]. A few previous studies attempted to spatialize R_0 _[10-12], mostly for diseases that are transmitted directly (e.g., foot-and-mouth disease, avian influenza). These studies identified high-risk areas based on landscape data and explored characteristics of epidemics, such as the spread distance, and the efficacy of control measures. Hartemink et al. [13] investigated a spatial R_0_at district level for the veterinary blue tongue disease in the Netherlands.

We also developed a spatially-explicit modelling approach to represent spatial variations in people-vector contacts at the landscape scale using multi-agent simulations (MAS) [14]. An agent is an autonomous computer entity capable of interacting with other agents and adapting its behaviour to a changing environment [15,16]. Agents can represent heterogeneous entities, e.g. people, animals, institutions, or land parcels, with their specific attributes and decision rules. The agent-based approach allows simulation and understanding of complex systems through the modelling of discrete events [17]. MAS can be used as a virtual laboratory to test hypotheses impossible to test in the field. MAS are particularly well suited to model disease systems as they combine biological, environmental and social processes.

Finally, we developed a spatially-explicit population dynamics model of mosquito populations [18,19], integrating the dynamics of their breeding sites (water bodies in which the females lay their eggs), the presence of hosts, and landscape attributes controlling the spread of mosquitoes. The model is mechanistic as it uses an *a priori *mathematical description of the main processes determining mosquito population dynamics. It is also deterministic as it represents an average behaviour of the population an approach that is well adapted for large populations, such as mosquito populations.

### Method to generalize across the case studies

The set of propositions described below were generated in an inductive manner, by generalisation from the set of empirical studies that we conducted. For each case study, we extracted the main conclusion(s) on the role of land use and land cover in the transmission cycle. The validity of these conclusions was then evaluated for each of the other case studies. In a synthesis table, we identified all the case studies for which a particular conclusion was empirically supported. We also identified disease systems for which the literature suggests that this conclusion may be valid.

### The eight case studies

We summarize below and in Table 1 the case studies reviewed here.

**Table 1:** Description of the eight case studies included in the review

	***WNV Senegal***	***TBE Latvia***	***Sandflies Pyrenees***	***RVF Senegal***	***WNF Camargue***	***PUUV Belgium***	***Lyme Belgium***	***Malaria Camargue***
*Pathogen*	flavivirus	flavivirus	protozoan parasite	phlebovirus	flavivirus	hantavirus	spirochetal bacteria	eukaryotic protist

*Vector or host*	mosquito	tick	sandfly	mosquito	mosquito	rodent	tick	mosquito

*Region*	Senegal river basin	Latvia/northeastern Latvia	French Pyrennees	Ferlo, Senegal	Camargue, France	Belgium	Belgium	Camargue, France

*Scale*	department	country	3 departments	department	ecounit	country	country	ecounit

*Spatial resolution*	30 m	100-30 m	30 m	2.4 m	30 m	30 m, municipality	municipality	30 m

*Climate*	semi-arid	temperate	mediterranean	semi-arid	mediterranean	temperate	temperate	mediterranean

*Field data*	horse serology	human cases	sandfly trapping	ruminant serology	horse & bird serology	rodent serology; human cases	human cases	mosquito trapping

*Analyses*	statistical	statistical	statistical	statistical; simulation model	simulation model	statistical	statistical	Multi-agent simulation

#### West Nile virus (WNV) transmission in the Senegal River basin

Chevalier et al. [20] conducted a serological study on horses in five ecologically contrasted regions of the Senegal River basin (Senegal) to assess WNV transmission. Blood samples were taken from 367 horses from the five regions and screened by ELISA for anti-WNV IgM and IgG. Positive samples were then confirmed by seroneutralization. The seroprevalence rate was 85% overall but it varied significantly between sites. To assess whether environmental conditions could explain these differences, a land cover map was derived from two satellite images from the dry and wet seasons, and the surface covered by each land-cover type was calculated for each study area. Environmental data were analysed using principal components analysis and generalized linear mixed models.

#### Tick-borne encephalitis (TBE) incidence in rural parishes of Latvia

Vanwambeke et al. [21] investigated the landscape-level factors influencing TBE incidence in rural parishes of Latvia, distinguishing between land cover, use and ownership. Land cover was used to depict the ecological suitability of the landscape for ticks and their hosts. Landscape composition and configuration were extracted from land cover maps. Land use represented human exposure to ticks, mostly through visits to forests. It was measured using proxy variables extracted from agricultural and household censuses. Land ownership of forests represented the accessibility to vector habitats for the public. Data were analysed using non-spatial and spatial negative binomial regression models.

#### Sandfly abundance in the French Pyrenees

A spatially-explicit R_0 _model was developed for canine leishmaniasis in the French Pyrennées [9]. An important variable for such a model is the density of the vector, which was estimated continuously in space using multivariate regression models. Based on extensive field trapping of sandflies, and using landscape composition and configuration at a medium spatial resolution and remotely sensed climate-related factors at a coarse spatial resolution, the abundance of sandflies was predicted throughout the study area. This was then used as an input for the calculation of a spatially-explicit R_0_.

#### Rift Valley Fever (RVF) in the semi-arid region of the Ferlo, Senegal

The impact of landscape variables on the transmission of RVF in small ruminants was investigated in the semi-arid region of Barkedji, Ferlo, Senegal [22]. The relationship between landscape features, derived from a very high spatial resolution image, and serologic incidence was analysed statistically using a mixed-effect logistic regression model. A model of mosquito population dynamics was also developed. This model, based on current knowledge on the biology of the two RVF vectors species, *Aedes vexans *and *Culex poicilippes*, takes into account the main events of the mosquito life cycle and climatic fluctuations. Simulations of daily spatial and temporal variations in the area of temporary ponds around a village in Senegal relied on the Tropical Rainfall Measuring Mission (TRMM) rainfall product [18,19]. Mosquito population dynamics was simulated based on variations in water level and surface.

#### Animal hosts of West Nile Fever (WNF) in the Camargue region

Based on the seasonal distribution of mosquito and bird populations, simulations of introduction, amplification and emergence of WNV under different realistic scenarios were produced and compared with seroprevalence measured in horse and bird populations [23].

#### Rodent-borne Puumala hantavirus (PUUV) in Belgium

The link between environmental features and PUUV prevalence in bank vole population was investigated in Belgium. Linard et al. [24] explored the relationship between environmental variables and host abundance, PUUV prevalence in the host, and human cases of nephropathia epidemica. Statistical analyses were carried out on 17 broadleaf forest sites. To understand causal pathways between environment and disease risk, the study distinguished between environmental factors related to the abundance of hosts, such as land-surface attributes, landscape configuration, and climate, and factors that may favour virus survival in the environment, such as climate and soil attributes. A national scale model explained the spatial distribution of PUUV human infections at the municipality level [25].

#### Geographic distribution of human cases of Lyme borreliosis (LB) in Belgium

The impact of fine-grained landscape patterns on the exposure of people to LB infection was also investigated in Belgium. A combination of factors linked to the vector and host populations, landscape attributes, and socio-economic factors were included in a negative binomial regression to explain the number of LB cases per municipality [25].

#### Risk of malaria re-emergence in the Camargue

**Figure 1:**
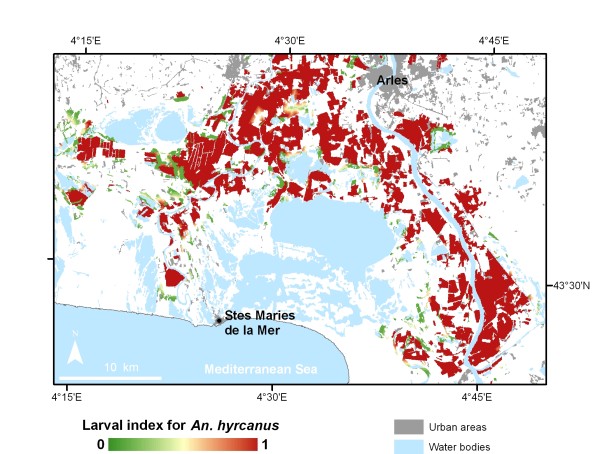
**Larval index map for the mosquito *Anopheles hyrcanus *derived from a statistical model associating entomological field data with satellite imagery in the Camargue, France**.

A larval index for the main potential vector of malaria in the Camargue area, *Anopheles hyrcanus*, was defined as the probability of observing its larvae in a given site at least once over a year. It was mapped by associating in a statistical model environmental indices that were derived from high spatial resolution imagery and entomological field data (Figure 1) [26]. Linard et al. [14] developed a spatially-explicit multi-agent simulation representing the spatio-temporal dynamics of interactions between the agents that could influence malaria transmission in the Camargue: people, mosquitoes, animal hosts and landscape. This model integrates movements and behaviours of people and vectors, and factors influencing transmission risk spatially. The model allowed testing potential drivers of malaria re-emergence such as changes in biological attributes of vectors, agricultural practices, land use, tourism activities and climate. Scenarios of possible futures varied the value of exogenous variables (e.g., tourist population visiting the area), initial conditions (e.g., land cover, in response to changes in land use policies or market forces) or parameters (e.g., level of protection of visitors against mosquito bites) [27].

### The ten propositions

#### 1. Landscape attributes may influence the level of transmission of an infection

**Figure 2:**
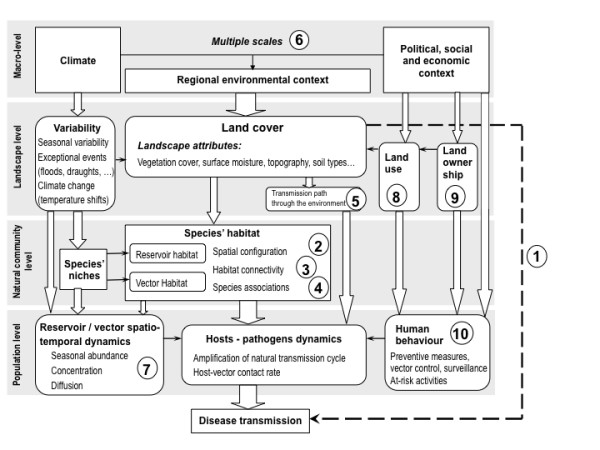
**Graphical representation of the landscape determinants of disease transmission**. The numbers refer to the ten propositions formulated in this paper.

This general proposition applies to all our studies (Table 2). The distribution, density, behaviour, and population dynamics of arthropod vectors and their non-human hosts are partially controlled by landscape features. The spatial distribution of vectors and the level of transmission is thus influenced by the environment [28,29]. This was illustrated by studies testing the relationship between disease occurrence and environmental features [30,31]. The case of WNV in Senegal illustrates this link, and the subsequent propositions provide details on the causal links between landscapes and diseases (Figure 2). In the serological study in the Senegal River basin, Chevalier et al. [20] found that IgG seroprevalence rate in horses was decreasing with proximity to seawater, flooded banks and salted mudflats. These landscape features acted as protective factors as they are highly unfavourable to the presence of *Culex *mosquitoes, the main WNV vectors. In Senegal, environmental constraints on vector distribution are more limitative for WNV transmission than bird distribution. Similar results were observed in the Camargue region, France and in Iowa, also with significant relationships between landscape features and seroprevalence of WNV [32,33], with different risk and protective factors though. This highlights the need for a landscape scale analysis of infections, especially when there are multiple possible hosts and vectors species.

**Table 2:** Validation of the ten propositions proposed in this paper with the eight case studies included in the review

	***WNV Senegal***	***TBE Latvia***	***Sandflies Pyrenees***	***RVF Senegal***	***WNF Camargue***	***PUUV Belgium***	***Lyme Belgium***	***Malaria Camargue***
*1. Landscape attributes*	E	E	+	E	E	E	E	+

*2. Spatial configuration*		E	E		+	E	E	

*3. Habitat connectivity*				E	+	E		

*4. Species associations*	+	+			E	+	+	

*5. Transmission paths*		+				E	+	

*6. Multiple scales*		E	E	+		E		

*7. Concentration, diffusion*				E	+			E

*8. Land use*		E				E	E	+

*9. Land ownership*		E						

*10. Human behaviour*		E				E	E	E

E: empirical evidence from that case study

+: case study is consistent with the proposition, based on the literature

-: case study is in disagreement the with proposition, based on the literature

#### 2. Spatial variations in disease risk depend not only on the presence and area of critical habitats but also on their spatial configuration

Numerous studies aimed at understanding associations between land cover and disease risk focus on the presence of critical habitats for vectors or reservoir hosts [34-40]. In most cases, the surface area of those habitats was used to quantify landscape characteristics, ignoring their spatial configuration. Yet, more complex and fragmented landscapes are associated with more ecotones (i.e., transition areas between two adjacent ecosystems), which increase the likelihood of contacts between species associated with various habitats [41]. Moreover, fragmented landscapes provide more habitats for edge species, and a greater diversity of resources. For example, ecotones between forests and open areas commonly have high tick densities [42], as well as higher incidence of infection [43]. The incidence of LB was significantly associated with the importance of land cover edges between forest and herbaceous land cover types in the US [44,45]. Allan et al. [46] found an increasing density of infected tick nymphs with decreasing size of forest patch. In Belgium, Linard et al. [25] showed that the probability of LB infection was higher in areas with a large interface between settlements and forests in peri-urban areas. In the multivariate statistical analysis of TBE incidence in rural parishes of Latvia [21], human cases of TBE were associated with the mean area of forest patches, their shape, and the proportion of mixed and transitional vegetation cover types around forests. Conversely, TBE incidence was lower not only where there were relatively large areas of unfavourable land covers for ticks, such as arable land, but also where forests were surrounded by agricultural land. Similar results were found in the statistical model predicting the abundance of the sandfly *Phlebotomus ariasi *in the French Pyrenees [9]. It was best predicted with landscape descriptors measured in a 1000-m buffer, including Shannon's landscape diversity index (i.e., a measure of the diversity and balance in landscape composition), the proportion of forests, and the mean area of forest patches. Others studies on different vector-borne and zoonotic diseases highlighted the importance of habitats configurations for infection transmission. For example, Pradier et al. [32] showed a significant association between WNV's level of circulation in Southern France and a landscape metrics measuring the degree of interweaving of land cover classes. Graham et al. [31] also demonstrated that habitat form was related to the prevalence in China of human alveolar echinococcosis caused by a helminth, the tapeworm *Echinococcus multilocularis*.

#### 3. Disease risk depends on the connectivity of habitats for vectors and hosts

**Figure 3:**
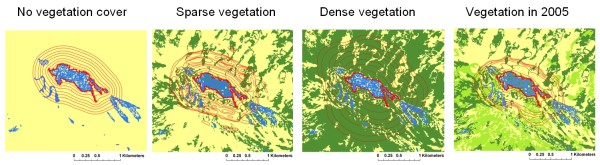
**Spread of the mosquito *Culex poicilipes *around a breeding site in different hypothetical landscapes in the semi-arid region of the *Ferlo*, Senegal, based on model simulations**. Red *isolines *depict the mosquito density. Vegetation is represented in green and bare soil in yellow.

The proximity of vector and host habitats may not result in a high level of risk if these critical habitats are not connected spatially by landscape features favourable to the circulation of vectors and/or hosts. Spatial diffusion of vectors is particularly crucial in the case of mosquito-borne diseases. At night, when female mosquitoes generally feed, most of the hosts (e.g., humans, cattle) are immobile. The ability of female vectors to spread from their breeding sites to hosts increases host/vector contacts. Landscape features largely control these movements [47]. The study of RVF in the semi-arid region of the Ferlo in Senegal showed that ruminant herds living around temporary water bodies were at greater risk of RVF if they were located close to ponds surrounded by vegetation [22]. A landscape closure index - representing the proportion of surface around each pond covered by vegetation such as dense forest and shrub savannah - was positively correlated with higher serologic incidence. The presence of dense vegetation around water bodies favours the spread of mosquitoes from the pond where they breed to the nearby ruminant herds (Figure 3). The importance of connectivity between habitats for the spread of mosquitoes was also demonstrated for *Culex *species in Southern France [48]. The connectivity between forest patches may also influence rodent populations and therefore the transmission of rodent-borne diseases such as hantavirus. Linard et al. [24] found that the spatial distribution of bank voles was different during epidemic and non-epidemic years. The number of bank voles captured was higher in more isolated forest patches during the non-epidemic year, whereas it was higher in less isolated patches during the epidemic year. Well-connected patches have more chances to be recolonized after local extinctions [49]. Habitat connectivity could also influence the virus occurrence in hosts by controlling movements of individuals and thus contact rate between infected and susceptible rodents. Langlois et al. [50] observed that hantavirus incidence in deer mice was higher in landscapes with a higher level of fragmentation of the preferred habitat. In Western Africa, Guerrini et al. [51] showed that the riverine forest fragmentation level is a critical factor to determine the habitat of riverine tsetse species, vectors of animal trypanosomosis.

#### 4. The landscape is a proxy for specific associations of reservoir hosts and vectors linked with the emergence of multi-host diseases

Great numbers of hosts and vectors species are potentially involved in the transmission of WNF, making its epidemiology complex. One should better understand underlying processes accounting for observed patterns of WNF when correlations between land use/cover and disease prevalence have been established [52]. In the Camargue region, we showed that epidemic processes of introduction, amplification and emergence of WNV were related to specific associations of hosts and vectors species in time and space. Based on maps of seasonal distributions of mosquito and bird populations, simulations of introduction, amplification and emergence of WNV were compared with seroprevalence measured in horse and bird populations [23]. Introduction of the virus by migratory birds explained accurately the observed spatial patterns of WNV transmission, whereas overwintering of WNV in mosquitoes did not. *Cx. modestus *was identified as the main amplifier of WNV, which is consistent with competence studies [53]. In the Camargue, the virus was only isolated in sparrows and magpies [54]. Yet, other competent bird species - or all bird species - may contribute to WNV amplification. The bird community composition did not seem to play a major role in the amplification of WNV in the Camargue (no "dilution effect"), unlike what was observed in the New World [52,55,56]. The final risk map, based on landscape attributes, synthesizes the different processes leading to WNV emergence in horses and identifies risk areas requiring veterinary surveillance.

#### 5. To understand ecological factors influencing spatial variations of disease risk, one needs to take into account the pathways of pathogen transmission between vectors, hosts, and the physical environment

The transmission of vector-borne diseases requires a direct contact - i.e., a bite - between an infectious vector and a susceptible host. By contrast, zoonoses such as PUUV can be transmitted directly, by physical contact between infected and susceptible hosts, or indirectly, with the environment as an intermediate. Actually, the virus can be shed in the environment via rodent excretions, and transmitted to humans in aerosols [57,58]. The environmental conditions controlling the direct and indirect transmission paths differ. In a study of the link between environment and PUUV prevalence in bank voles in Belgium, Linard et al. [25] showed that PUUV prevalence among bank voles is more linked to variables favouring the survival of the virus in the environment, and thus indirect transmission. In particular, low winter temperatures were strongly linked to high prevalence among bank voles, and high soil moisture was associated with high numbers of nephropathia epidemica cases among humans. The risk of transmission to humans is therefore not only determined by host abundance, but also by the indirect transmission path, largely controlled by climatic factors and soil characteristics influencing virus survival in the environment. Another illustration is provided by the differences in dynamics of two pathogens vectored by the same species, *Borrelia spp*. and TBE virus. Transmission to ticks related to systemic and non-systemic host infections (respectively, infection of many and specific parts of the body) has been described for both pathogens. Yet, transmission through co-feeding of ticks in non-systemically infected hosts may be more important to sustain the transmission of TBE [59,60]. As a result, spatial variations of TBE infection risk are controlled by more restrictive ecological conditions, as closer interactions between infected competent hosts and ticks are required compared to LB. The TBE transmission cycle is therefore more fragile [61,62]. The transmission via the environment can also play a role in the transmission of avian influenza viruses: contaminated water supplies can constitute a reservoir that maintains the virus in the environment and be a source of contamination for wild or domestic birds [63].

#### 6. The emergence and distribution of infection through time and space is controlled by different factors acting at multiple scales

The emergence of a disease in a particular region is associated with multiple macro-level changes such as a shift in political and economic regimes that influence people's livelihood strategies and therefore their interaction with natural ecosystems [64,65], rapid conversion of natural habitats, and urbanization. In some cases, variations in climate may act synergistically with these socio-economic changes, even though the relative magnitude of climate change impacts remains contentious [66]. Macro-economic and climatic conditions create favourable or unfavourable background conditions for the development of pathogens, vectors, and hosts. The actual realisation of the transmission cycle and transmission to humans depends on the overlap of the preferred habitats of competent vectors and infected hosts, and on the entry of humans into these infected areas. Therefore, the fine-grained spatial heterogeneity in disease emergence is determined by natural and cultural landscape attributes that act as fine-scale spatial determinants of the multiple factors controlling the transmission cycle. For example, while an acceleration of warming of spring temperatures in the Baltic region played some role in the dramatic increase in TBE incidence [67], it did not account for the high spatial heterogeneity of incidence at an infra-national scale. Landscape factors related to the ecology of the disease and to human activities better explain this spatial heterogeneity [21]. In Western Europe, bank voles are known to display cyclic abundance peaks, which significantly increase PUUV infection among rodents and humans. These peaks are related to high tree seed production, triggered by high summer and autumn temperatures the preceding years [68,69]. While such macro-scale factors influence PUUV incidence at the regional scale, landscape characteristics related to rodent habitats and human land use determine the local scale spatial heterogeneity of transmission. Spatial variations in sandfly abundance in the French Pyrenees was also explained by a combination of coarse and fine resolution remotely-sensed metrics that are associated with, respectively, regional climatic trends and local-scale landscape variables reflecting land use [9]. In (disease) ecology, the relevance of the scale factor has been widely demonstrated [70] and yet it remains poorly explored in empirical studies of landscape epidemiology.

#### 7. Landscape and meteorological factors control not just the emergence but also the spatial concentration and spatial diffusion of infection risk

In the semi-arid region of the Ferlo, Senegal, temporary ponds - i.e., that are flooded after the first rains and remain dry for most of the dry season - constitute a favourable habitat for RVF vectors, *Ae. vexans *and *Cx. poicilippes*. Modelling spatial and temporal variations in water level and surface of temporary ponds around a village in Senegal allowed simulating mosquito population dynamics and host distribution [18,19]. Results showed that rainfall drives *Culex *and *Aedes *populations and therefore the risk of circulation of RVF virus. Depending on the frequency of rainfall events, high densities of *Culex *and *Aedes *may occur simultaneously. This co-occurrence of species leads to a higher risk of transmission, *Ae. vexans *and *Cx. poicilipes *acting as, respectively, initiators and amplifiers of RVF virus circulation. Thus, in the Ferlo region, a combination of rainfall frequency and amount identifies years at risk for RVF. Landscape features also influence the spread distance of mosquitoes away from their breeding sites [19]. In East Africa, heavy rainfall events were associated with RVF outbreaks [71]. Such events lead to major increase in mosquito vector populations [72]. The flooded areas that are potential mosquito breeding sites ("dambos") constitute the main risk areas [73]. Meteorological conditions can also influence exposure of humans [74].

#### 8. Spatial variation in disease risk depends not only on land cover but also on land use, via the probability of contact between, on one hand, human hosts and, on the other hand, infectious vectors, animal hosts or their infected habitats

Land cover is defined by the attributes of the earth's land surface and immediate subsurface, including biota, soil, topography, surface and groundwater, and human structures. Land use is defined by the purposes for which humans exploit the land cover. While a focus on land cover helps understanding the presence of vectors and hosts, a focus on land use identifies which places people visit for specific activities, at what time of the day and of the year, and at what frequency. The attractivity of various places for a given activity depends on their attributes such as accessibility and value for that activity - e.g., presence of recreational features, easily accessible forests with amenities such as good trails. Our studies on human cases of PUUV and LB in Belgium showed that the spatial distributions of the two diseases were very dissimilar [25]. PUUV was mostly prevalent in forested, rural and low income municipalities as transmission to humans was mostly associated with hunting and forestry work. By contrast, LB was mostly found in forested, peri-urban and wealthy municipalities, as infection was mostly associated with gardening and recreational activities in forests. Thus, it is less land cover (i.e., the presence of broad-leaved forests) than land use (associated with income level and type of settlements) that controls the spatial distribution of human cases of these two diseases. Agents involved in different land uses - e.g., a tourist and a local farmer - also have varying levels of awareness of the risk of infection transmission and rate of adoption of preventive measures. Socio-economic factors such as income and education have been quantitatively related to frequency of human visits to forests and exposure to tick bites [75]. In Latvia, the main reasons for people to enter forests included looking for alternative livelihood sources or recreation. People with low income and education visit forests more commonly with the purpose of collecting wild food, while more wealthy and educated people are more likely to visit forests for recreation [75]. In our study of TBE incidence in rural parishes of Latvia [21], human cases of TBE were positively related to variables indicating wild food pickers and negatively related to variables associated with hikers. Different socio-economic groups are represented in various proportions in different regions of a country. In the Camargue, the scenarios tested using a multi-agent simulation showed that water management practices in rice fields influence the rate of contact between people and potential malaria vectors [27]. According to the flooding date of rice fields, the maximum abundance of vectors and people can be reached simultaneously, resulting in high contact rates. In a study on West Nile Virus disease risk in the US, Winters et al. [76] emphasized the importance of taking into account human activities to assess risk of vector exposure in montane areas heavily used for recreation in the summer.

#### 9. The relationship between land use and the probability of contact between vectors and animal hosts and human hosts is influenced by land ownership

Land ownership and access rules determine whether distributions of ticks and human activities overlap in space and time. In most countries, State forests are more accessible to the public than privately-owned forests. In the statistical analysis of TBE incidence in rural parishes of Latvia [21], land ownership proved to be an important explanatory variable, with a positive relationship between TBE incidence and the presence of State-owned forests. Because forest ownership is associated with different forest management practices, land ownership may reflect not just access to forests but also differences in forest management practices. The latter have an impact on habitat quality for ticks and tick-hosts, and on the attractiveness of forests for human activities. For example, forest age, use of plantation and sanitary cutting may affect the presence of ticks, rodents, and forest food. While issues of land ownership have been widely studied in conservation science [77], they have been ignored in landscape epidemiology.

#### 10. Human behaviour is a crucial controlling factor of vector-human contacts, and of infection

Humans can adopt a range of preventive measures to decrease the risk of contact with vectors or pathogens. The rate of adoption of these measures depends on the availability of such preventive measures, the nuisance of the vector, the risk of infection and its level of perception by susceptible agents. For transmission to occur, one needs a combination of a conducive environment, which is created by an ecosystem favourable to interactions between pathogens, vectors and their animal hosts, and risk behaviours by human hosts that lead to a greater incidence among a human population. For example, the emergence of TBE in the Baltics was associated with a complex combination of abiotic, biotic and human factors [64,75]. Similarly, the spatial distributions of PUUV and LB infections in Belgium were related to human activities and behaviours, among other factors [25]. In an area of Sweden endemic for TBE and LB, differences were found in adoption of preventive measures between permanent and part-time residents and between genders, suggesting differing risk perception [78]. Dengue infection in northern Thailand was explained by variables related to the ecology of *Aedes*, the mosquito vector, and by risk and protective behaviours [79,80]. One of the greatest challenges in landscape epidemiology is to better integrate human behaviours.

## Discussion

The above propositions have the status of hypotheses requiring further testing in different geographic and biological contexts. Yet, they further our understanding of the impact of land-use and land-cover change on the transmission cycle of vector-borne diseases. A practical implication of this enhanced knowledge is to identify indicators of "pathogenic landscapes", to provide early warning signals of an increased likelihood of disease transmission. A static view of the "pathogenicity" of landscapes overlays maps of the spatial distribution of: (i) vectors and their habitats, (ii) animal hosts carrying specific pathogens and their habitat, (iii) susceptible human hosts and their land use. A more dynamic view would represent the spatial and temporal dynamics of these agents at multiple scales. Potential indicators of a higher transmission risks would be derived from any combination of social and ecological processes that are associated with spatial boundaries and temporal transitions that increase - or create new - interactions between disease transmission agents. Spatial indicators of infection risks would be associated with ecotones; margins of the geographic distributions of vectors, hosts or pathogens; and highly connected places, being at the crossroad of circulating vectors, hosts or pathogens. Temporal indicators of infection risks would be associated with biological mutation of pathogens, invasion of vectors, change in composition of animal host population, migration of naïve human hosts, abrupt land use/cover changes (not just land cover conversions but also subtle land cover modifications), political and economic changes, rapid climatic changes... Importantly, it is synergistic interactions between several of these indicators that are most likely to be associated with high infection risks.

The above findings have implications for the design of public health surveillance systems. For example, georeferencing of serological data collected on hosts is essential. For human cases, recording the place of infection in addition to the place of residence of patients is also necessary. Systematic trapping of vectors and/or animal hosts for serological surveys should be spatially targeted on areas with a high risk of transmission. This could be achieved through spatially stratified sampling schemes defined based on the relevant landscape attributes. Budgeting should allow for rapidly increasing the intensity of serological surveys on hosts in periods of unexpected socio-economic, ecological or climatic transitions that have the potential to affect transmission of infections. Data on land cover, land use, and socio-economic factors associated with risk behaviours should be systematically integrated in surveillance systems. Finally, the ability of public health analysts to integrate data from different sources, at multiple scales, and representing various components of disease systems should be enhanced.

The different modelling approaches used in the case studies were highly complementary. A detailed and accurate land cover mapping by remote sensing was at the basis of the methods used. Spatial statistical models were used to explore empirical relationships between disease systems and landscape patterns, as a preliminary approach of disease systems for the development of more integrated models. The finding of statistically significant associations does not establish causal relationships. Moreover, regression models cannot be used for extrapolation beyond the region of the variable space corresponding to the original data. R_0 _models allow investigation of establishment potential in the absence of a disease. They are based on a representation of biological processes of transmission. Yet, the spatial heterogeneity of environmental attributes is not explicitly included in these models. The development of multi-agent simulations necessitates a considerable amount of data and a good preliminary knowledge of the disease system. MAS represent the dynamics of people-vector contacts in space and time and are therefore ideal to explore scenarios associated with conditions that have not been observed previously. Coupling R_0 _models with MAS could therefore better represent the influence of spatial and temporal heterogeneity in people-vector contacts on the risk of establishment of an infection. There is a logical sequence in the use of the various methods, from the land cover description by remote sensing to the spatially-explicit multi-agent simulation, transiting through exploratory statistical analyses to better understand the key components of disease transmission and their associations.

## Conclusion

We conclude as we started, with a quote by E.N. Pavlovsky (1966):

"The epidemiological significance of a locality is determined by the following factors: (a) the landscape of an area with natural foci of diseases in newly-settled and reclaimed regions (...); (b) the extent and nature of contact between man and his natural environment."

All our case studies show that spatial variations in infection risk are controlled by three sets of factors: (i) the pathogenic cycle and the biology of vectors, hosts and pathogens; (ii) ecosystem processes at the landscape scale, as influenced by ecosystem structure and composition, landscape connectivity and configuration, climate, species interactions; and (iii) land use, human behaviour and mobility, knowledge and perception of disease risk, and socio-economic conditions. In general, previous studies in spatial epidemiology have ignored or given less emphasis to the latter set of factors (i.e., land use and human behaviours). The challenge in landscape epidemiology is to integrate dynamically these different factors, with an emphasis on their interactions and not just on their spatial overlay. By identifying a set of propositions on factors controlling these interactions, this review contributes to a general understanding of spatial variations in disease risk.

## Competing interests

The authors declare that they have no competing interests.

## Authors' contributions

EFL designed and coordinated the synthesis study. AT, SV, CL, VS contributed to the synthesis study and analyzed the data for individual case studies. All authors wrote, read and approved the final manuscript.

## References

[B1] Patz JA Daszak P Tabor GM Aguirre AA Pearl M Epstein J Wolfe ND Kilpatrick AM Foufopoulos J Molyneux D Bradley D Members of the Working Group on Land Use Change Disease Emergence. Unhealthy landscapes: policy recommendations on land use change and infectious disease emergence Environmental Health Perspectives 2004 112 1092 1098 10.1289/ehp.6877 15238283 PMC1247383

[B2] Ostfeld RS Glass GE Keesing F Spatial epidemiology: an emerging (or re-emerging) discipline Trends in Ecology & Evolution 2005 20 6 328 336 10.1016/j.tree.2005.03.009 16701389

[B3] May JM History, definition, and problems of medical geography: a general review XVIIth International Geographical Congress, Washington. International Geographical Union 1952 1 9 10.1016/s0277-9536(78)80009-4 310579

[B4] Pavlovsky EN Natural nidality of transmissible diseases in relation to the landscape epidemiology of zooanthroponoses 1966 Moscow: Peace Publishers

[B5] McDonald G The epidemiology and control of malaria 1957 London: Oxford University Press

[B6] Rogers DJ The dynamics of vector-transmitted diseases in human communities Philosophical Transactions of the Royal Society of London. Series B, Biological Sciences 1988 321 1207 513 539 10.1098/rstb.1988.0106 2907156

[B7] Anderson RM May RM Infectious diseases of humans: dynamics and control 1991 Oxford University Press

[B8] Ponçon N Tran A Toty C Luty AJ Fontenille D A quantitative risk assessment approach for mosquito-borne diseases: malaria re-emergence in southern France Malar J 2008 7 147 10.1186/1475-2875-7-147 18673551 PMC2527012

[B9] Hartemink NA Vanwambeke SO Heesterbeek JAP Rogers DJ Morley D Lambin EF Pesson B Davies C Mahamdallie S Ready P Hartemink NA Modelling and mapping the basic reproduction number R_0 _for canina leishmaniasis: a case study for a region in South West France Vector-borne diseases: the basic reproduction number R0 and risk maps.PhD Thesis 2009 University of Utrecht

[B10] Ferguson NM Donnelly CA Anderson RM The foot-and-mouth epidemic in Great Britain: pattern of spread and impact of interventions Science 2001 292 1155 60 10.1126/science.1061020 11303090

[B11] Boender GJ Hagenaars TJ Bouma A Nodelijk G Elbers AR De Jong MC Van Boven M Risk maps for the spread of highly pathogenic avian influenza in poultry PLoS Comput Biol 2007 3 e71 10.1371/journal.pcbi.0030071 17447838 PMC1853123

[B12] Curtis A Mills JW Blackburn JK 2007. A spatial variant of the Basic Reproduction Number for the New Orleans yellow fever epidemic of 1878 The Professional Geographer 2007 59 492 502 10.1111/j.1467-9272.2007.00637.x

[B13] Hartemink NA Purse BV Meiswinkel R Brown HE De Koeijer A Elbers ARW Boender GJ Rogers DJ Heesterbeek JAP Mapping the basic reproduction number (R_0_) for vector-borne diseases: a case study on bluetongue virus Epidemics 2009 1 153 161 10.1016/j.epidem.2009.05.004 21352762

[B14] Linard C Ponçon N Fontenille D Lambin EF A multi-agent simulation to assess the risk of malaria re-emergence in southern France Ecological Modelling 2009 220 2 160 174 10.1016/j.ecolmodel.2008.09.001

[B15] Bousquet F Barreteau O Le Page C Mullon C Weber J An environmental modelling approach: The use of multi-agents simulations Advances in environmental and ecological modelling 1999 Paris: Elsevier 113 122

[B16] Hare M Deadman P Further towards a taxonomy of agent-based simulation models in environmental management Mathematics and Computers in Simulation 2004 64 25 40 10.1016/S0378-4754(03)00118-6

[B17] Bousquet F Le Page C Multi-agent simulations and ecosystem management: a review Ecological Modelling 2004 176 313 332 10.1016/j.ecolmodel.2004.01.011

[B18] Soti V Tran A Bailly JS Puech C Lo Seen D Begue A Assessing optical Earth observation systems for mapping and monitoring temporary ponds in arid areas International Journal of Applied Earth Observation and Geoinformation 2009 11 344 351 10.1016/j.jag.2009.05.005

[B19] Soti V Puech C Lo Seen D Bertran A Vignolles C Mondet B Dessay N Tran A The potential of remote sensing and hydrologic modelling to assess the spatio-temporal dynamics of ponds in the Ferlo region (Senegal) Hydrology and Earth System Sciences 2010 14 4 16 10.5194/hess-14-1449-2010

[B20] Chevalier V Dupressoir A Tran A Diop OM Gottland C Diallo M Etter E Ndiaye M Grosbois V Dia M Gaidet-Drapier N Sall AA Soti V Niang M 2010. Environmental risk factors of West Nile infection in the Senegal River basin Epidemiology and infection 2010 138 1601 1609 10.1017/S095026881000035X 20175940

[B21] Vanwambeke SO Šumilo D Bormane A Lambin EF Randolph SE Landscape predictors of tick-borne encephalitis in Latvia: land cover, land use, and land ownership Vector-borne and Zoonotic Diseases 2010 10 497 506 10.1089/vbz.2009.0116 19877818

[B22] Maura J Application de la télédétection à très haute résolution spatiale à l'étude d'une maladie à transmission vectorielle: Mise en relation de variables paysagères et de l'incidence sérologique sur ovins pour l'identification de zones à risque de transmission de la Fièvre de la Vallée du Rift 2007 Montpellier: CIRAD report

[B23] Tran A Gaidet N L'Ambert G Balenghien T Balanca G Chevalier V Soti V Ivanes C Etter E Schaffner F Baldet T De La Rocque S The use of remote sensing for the ecological description of multi-host disease systems: a case study on West Nile virus in southern France Veterinaria Italiana 2007 43 3 687 697 20422548

[B24] Linard C Tersago K Leirs H Lambin EF Environmental conditions and Puumala virus transmission in Belgium International Journal of Health Geographics 2007 6 55 10.1186/1476-072X-6-55 PMC2234401 18078526

[B25] Linard C Lamarque P Heyman P Ducoffre G Luyasu V Tersago K Vanwambeke SO Lambin EF Determinants of the geographic distribution of Puumala virus and Lyme borreliosis infections in Belgium International Journal of Health Geographics 2007 6 15 10.1186/1476-072X-6-15 PMC1867807 17474974

[B26] Tran A Ponçon N Toty C Linard C Guis H Ferre J-B Lo Seen D Roger F De La Rocque S Fontenille D Baldet T Use of remote sensing to map larval and adult populations of *Anopheles hyrcanus *(Diptera: Culicidae) a potential malaria vector in Southern France International Journal of Health Geographics 2008 7 9 18302749 10.1186/1476-072X-7-9 PMC2291038

[B27] Linard C Ponçon N Fontenille D Lambin EF Risk of malaria re-emergence in southern France: testing scenarios with a multi-agent simulation model EcoHealth 2009 6 1 135 147 10.1007/s10393-009-0236-y 19449076

[B28] Patz JA Graczyk TK Geller N Vittor AY Effects of environmental change on emerging parasitic diseases International Journal of Parasitology 2000 30 1395 405 10.1016/S0020-7519(00)00141-7 11113264

[B29] Norris D Mosquito-borne diseases as a consequence of land use change EcoHealth 2004 1 19 24 10.1007/s10393-004-0008-7

[B30] Boone JD McGwire KC Otteson EW Debaca RS Kuhn EA Villard P Brussard PF St Jeor SC Remote Sensing and Geographic Information Systems: Charting Sin Nombre Virus Infections in Deer Mice Emerging Infectious Diseases 2000 6 248 258 10.3201/eid0603.000304 10827114 PMC2640872

[B31] Graham AJ Danson FM Giraudoux P Craig PS Ecological epidemiology: landscape metrics and human alveolar echinococossis Acta Tropica 2004 91 267 278 10.1016/j.actatropica.2004.05.005 15246932

[B32] Pradier S Leblond A Durand B Biodiversity landscape metrics and West Nile virus circulation in southern France Vector Borne and Zoonotic Diseases 2008 8 253 263 10.1089/vbz.2007.0178 18429693

[B33] Degroote JP Sugumaran R Brend SM Tucker BJ Bartholomay LC Landscape, demographic, entomological, and climatic associations with human disease incidence of West Nile virus in the state of Iowa, USA Int J Health Geogr 2008 7 19 18452604 10.1186/1476-072X-7-19 PMC2396613

[B34] Beck LR Rodriguez MH Dister SW Rodriguez AD Rejmankova E Ulloa A Meza RA Roberts DR Paris JF Spanner MA Washino RK Hacker C Legters LJ Remote Sensing as a landscape epidemiologic tool to identify villages at high risk for malaria transmission Am J Trop Med Hyg 1994 51 271 280 7943544 10.4269/ajtmh.1994.51.271

[B35] Rejmankova E Roberts DR Pawley A Manguin S Polanco J Predictions of adult anopheles albimanus densities in villages based on distances to remotely sensed larval habitats Am J Trop Med Hyg 1995 53 482 488 7485706 10.4269/ajtmh.1995.53.482

[B36] Roberts DR Paris JF Manguin S Harbach RE Woodruff R Rejmankova E Polanco J Wullschleger B Legters LJ Predictions of malaria vector distribution in Belize based on multispectral satellite data Am J Trop Med Hyg 1996 54 304 308 8600771 10.4269/ajtmh.1996.54.304

[B37] Sharma VP Dhiman RC Ansari MA Nagpal BN Srivastava A Manavalan P ADIGA S Radhakrishnan K Chanrasekhar MG Study of the feasibility of delineating mosquitogenic conditions in and around Delhi using Indian Remote Sensing satellite data Indian Journal of Malariology 1996 33 107 125 9014394

[B38] Malone JB Abdel-Rahman MS El Bahy MM Huh OK Shafik M Bavia M Geographic Information Systems and the distribution of Schistosoma mansoni in the Nile Delta Parasitology Today 1997 13 112 119 10.1016/S0169-4758(97)01009-0 15275115

[B39] Eisen RJ Eisen L Lane RS Remote sensing (Normalized difference vegetation index) classification of risk versus minimal risk habitats for human exposure to Ixodes pacificus (Acari: Ixodidae) nymphs in Mendocino County, California Journal of Medical Entomology 2005 42 75 81 10.1603/0022-2585(2005)042[0075:RSNDVI]2.0.CO;2 15691012

[B40] Minakawa N Munga S Atieli F Mushinzimana E Zhou GF Githekp AK Yan GY Spatial distribution of anopheline larval habitats in Western Kenyan highlands: Effects of land cover types and topography American Journal of Tropical Medicine and Hygiene 2005 73 157 165 16014851

[B41] Despommiers D Ellis BR Wilcox BA The role of ecotones in emerging infectious diseases EcoHealth 2006 3 281 289 10.1007/s10393-006-0063-3

[B42] Daniel M Kolar J Zeman P Pavelka K Sadlo J Predictive map of Ixodes ricinus high-incidence habitats and a tick-borne encephalitis risk assessment using satellite data Experimental & Applied Acarology 1998 22 417 433 10.1023/a:1006030827216 9680691

[B43] Kantso B Svendsen CB Jensen PM Vennestrom J Krogfelt KA Seasonal and habitat variation in the prevalence of Rickettsia helvetica in Ixodes ricinus ticks from Denmark Ticks and tick-borne diseases 2010 1 101 103 10.1016/j.ttbdis.2010.01.004 21771515

[B44] Jackson LE Hilborn ED Thomas JC Towards landscape design guidelines for reducing Lyme disease risk International Journal of Epidemiology 2006 35 315 322 10.1093/ije/dyi284 16394113

[B45] Horobik V Keesing F Ostfeld RS Abundance and Borrelia burgdorferi-infection prevalence of nymphal Ixodes scapularis ticks along forest-field edges EcoHealth 2006 3 4 262 268 10.1007/s10393-006-0065-1

[B46] Allan BF Keesing F Ostfeld RS Effect of forest fragmentation on Lyme disease risk Conservation Biology 2003 17 1 267 272 10.1046/j.1523-1739.2003.01260.x

[B47] Raffy M Tran A On the dynamics of flying insects populations controlled by large scale information Theoretical Population Biology 2005 68 91 104 10.1016/j.tpb.2005.03.005 16023689

[B48] Balenghien T De l'identification des vecteurs du virus West Nile à la modélisation du risque d'infection dans le sud de la France PhD Thesis 2006 University of Grenoble

[B49] Kozakiewicz M Apeldoorn RV Bergers P Gortat T Kozakiewicz A Landscape approach to bank vole ecology Polish Journal of Ecology 2000 48 suppl 149 161

[B50] Langlois JP Fahrig L Merriam G Artsob H Landscape structure influences continental distribution of hantavirus in deer mice Landscape Ecology 2001 16 255 266 10.1023/A:1011148316537

[B51] Guerrini L Bord JP Ducheyne E Bouyer J Fragmentation analysis for prediction of suitable habitat for vectors: Example of riverine tsetse flies in Burkina Faso Journal of medical entomology 2008 45 1180 1186 10.1603/0022-2585(2008)45[1180:FAFPOS]2.0.CO;2 19058646

[B52] Ezenwa VO Godsey MS King RJ Guptill SC Avian diversity and West Nile virus: testing associations between biodiversity and infectious disease risk Proceedings of the Royal Society B-Biological Sciences 2006 273 109 117 10.1098/rspb.2005.3284 PMC1560012 16519242

[B53] Balenghien T Vazeille M Grandadam M Schaffner F Zeller H Reiter P Sabatier P Fouque F Bicout DJ Vector competence of some French Culex and Aedes mosquitoes for West Nile virus Vector Borne and Zoonotic Diseases 2008 8 589 95 10.1089/vbz.2007.0266 18447623

[B54] Jourdain E Schuffenecker I Korimbocus J Reynard S Murri S Kayser Y Gauthier-Clerc M Sabatier P Zeller HG West Nile virus in wild resident birds, Southern France, 2004 Vector Borne Zoonotic Dis 2007 7 448 52 10.1089/vbz.2006.0592 17767404

[B55] Swaddle JP Calos SE Increased avian diversity is associated with lower incidence of human West Nile infection: observation of the dilution effect PLoS One 2008 3 e2488 10.1371/journal.pone.0002488 PMC2427181 18575599

[B56] Allan BF Langerhans RB Ryberg WA Landesman WJ Griffin NW Katz RS Oberle BJ Schutzenhofer MR Smyth KN De St Maurice A Clark L Crooks KR Hernandez DE McLean RG Ostfeld RS Chase JM Ecological correlates of risk and incidence of West Nile virus in the United States Oecologia 2009 158 699 708 10.1007/s00442-008-1169-9 18941794

[B57] Kallio ER Klingstrom J Gustafsson E Manni T Vaheri A Henttonen H Vapalahti O Lundkvist A Prolonged survival of Puumala hantavirus outside the host: evidence for indirect transmission via the environment Journal of General Virology 2006 87 8 2127 2134 10.1099/vir.0.81643-0 16847107

[B58] Sauvage F Langlais M Yoccoz NG Pontier D Modelling hantavirus in fluctuating populations of bank voles: the role of indirect transmission on virus persistence Journal of Animal Ecology 2003 72 1 13 10.1046/j.1365-2656.2003.00675.x

[B59] Randolph SE Gern L Nuttall PA Co-feeding ticks: epidemiological significance for tick-borne pathogen transmission Parasitology Today 1996 12 472 479 10.1016/S0169-4758(96)10072-7 15275266

[B60] Randolph SE Gern L Co-feeding transmission and its contribution to the perpetuation of the Lyme Disease Spitochete Borrelia afzelii Emerging Infectious Diseases 2003 9 893 894 12899145 10.3201/eid0907.030116 PMC3023430

[B61] Randolph SE Šumilo D Takken W, Knols BGJ Tick-borne encephalitis in Europe: dynamics of changing risk Emerging pests and vector-borne diseases in Europe 2007 Wageningen: Wageningen Academic Publishers;

[B62] Hartemink NA Randolph SE Davis SA Heesterbeek JAP The basic reproduction number for complex disease systems: Defining R_0 _for tick-borne infections The American Naturalist 2008 171 743 754 10.1086/587530 18462128

[B63] Markwell DD Shortridge KF Possible waterborne transmission and maintenance of influenza viruses in domestic ducks Applied and Environmental Microbiology 1982 43 110 5 7055370 10.1128/aem.43.1.110-115.1982 PMC241789

[B64] Randolph SE Andolph SE Tick-borne encephalitis incidence in Central and Eastern Europe: consequences of political transition Microbes and Infections 2008 10 209 216 10.1016/j.micinf.2007.12.005 18316221

[B65] Randolph SE Tick-borne encephalitis virus, ticks and humans: short-term and long-term dynamics Current Opinion in Infectious Diseases 2008 21 462 467 10.1097/QCO.0b013e32830ce74b 18725794

[B66] Lafferty KD The ecology of climate change and infectious diseases Ecology 2009 90 888 900 10.1890/08-0079.1 19449681

[B67] Šumilo D Asokliene L Bormane A Vasilenko V Golovljova I Randolph SE Climate change cannot explain the upsurge of tick-borne encephalitis in the Baltics PLoS One 2007 2 e500 10.1371/journal.pone.0000500 PMC1876807 17551580

[B68] Tersago K Verhagen R Servais A Heyman P Ducoffre G Leirs H Hantavirus disease (nephropathia epidemica) in Belgium: Effects of tree seed production and climate Epidemiology and Infection 2009 137 250 256 10.1017/S0950268808000940 18606026

[B69] Clement J Vercauteren J Verstraeten WW Ducoffre G Barrios JM Vandamme A-M Maes P Van Ranst M Relating increasing hantavirus incidences to the changing climate: the mast connection International Journal of Health Geographics 2009 8 1 19149870 10.1186/1476-072X-8-1 PMC2642778

[B70] Horwitz P Wilcox BA Parasites, ecosystems and sustainability: an ecological and complex systems perspective International Journal for Parasitology 2005 35 725 732 10.1016/j.ijpara.2005.03.002 15925596

[B71] Linthicum KJ Anyamba A Tucker CJ Kelley PW Myers MF Peters CJ Climate and satellite indicators to forecast Rift Valley fever epidemics in Kenya Science 1999 285 397 400 10.1126/science.285.5426.397 10411500

[B72] Davies FG Linthicum KJ James AD Rainfall and epizootic Rift Valley fever World Health Org Rep 1985 63 941 943 PMC2536443 3879206

[B73] Pope KO Sheffner EJ Linthicum KJ Bailey CL Logan TM Kasischke ES Birney K Njogu AR Roberts CR Identification of Central Kenyan Rift Valley Virus vector habitats with Landsat TM and evaluation of their flooding status with airborne imaging radar Remote Sensing of Environnement 1992 40 185 196 10.1016/0034-4257(92)90002-2

[B74] Randolph SE Asokliene L Avsic-Zupanc T Bormane A Burri C Gern L Golovljova I Hubalek Z Knap N Kondrusik M Kupca A Pejcoch M Vasilenko V Zygutiene M Variable spikes in tick-borne encephalitis incidence in 2006 independent of variable tick abundance but related to weather Parasites & Vectors 2008 1 44 10.1186/1756-3305-1-44 PMC2614985 19068106

[B75] Šumilo D Bormane A Asokliene L Vasilenko V Golovljova I Avsic-Zupanc T Hubalek Z Randolph SE Socio-economic factors in the differential upsurge of tick-borne encephalitis in Central and Eastern Europe Reviews in Medical Virology 2008 18 81 95 10.1002/rmv.566 18183571

[B76] Winters AM Bolling BG Beaty BJ Blair CD Eisen RJ Meyer AM Pape WJ Moore CG Eisen L Combining mosquito vector and human disease data for improved assessment of spatial West Nile virus disease risk American Journal of Tropical Medicine and Hygiene 2008 78 654 665 18385365

[B77] Rissman AR Merenlender AM The conservation contributions of conservation easements: analysis of the San Francisco Bay Area protected land database Ecology and Society 2008 13 40

[B78] Stjernberg L Berglund J Tick prevention in a population living in highly endemic area Scandinavian Journal of Public Health 2005 33 432 438 10.1080/14034940510005932 16332608

[B79] Van Bethem BHB Vanwambeke SO Khantikul N Burghoorn-Maas C Panart K Oskam L Lambin EF Somboon P Spatial patterns of and risk factors for seropositivity fore dengue infection American Journal of Tropical Medicine and Hygiene 2005 72 201 208 15741558

[B80] Vanwambeke SO Van Bethem BHB Khantikul N Burghoorn-Maas C Panart K Oskam L Lambin EF Somboon P Multi-level analyses of spatial and temporal determinants for dengue infection International Journal of Health Geographics 2006 5 5 10.1186/1476-072X-5-5 16420702 PMC1373612

